# Sleep medication and melatonin use among Norwegian nurses – A cross‐sectional study

**DOI:** 10.1002/nop2.1057

**Published:** 2021-09-17

**Authors:** Ingeborg Forthun, Siri Waage, Ståle Pallesen, Bente Elisabeth Moen, Bjørn Bjorvatn

**Affiliations:** ^1^ Department of Global Public Health and Primary Care University of Bergen Bergen Norway; ^2^ Norwegian Competence Center for Sleep Disorders Haukeland University Hospital Bergen Norway; ^3^ Department of Psychosocial Science University of Bergen Bergen Norway; ^4^ Optentia, The Vaal Triangle Campus of the North‐West University Vanderbijlpark South Africa

**Keywords:** occupational health, shift work, sleep, sleep medication use

## Abstract

**Aim:**

To estimate the prevalence of sleep medication and melatonin use among nurses and to assess if factors related to work, sleep or mental health, were associated with such use.

**Design:**

A cross‐sectional study.

**Methods:**

A questionnaire survey including 2,798 Norwegian nurses. Associations were estimated using a modified Poisson regression model.

**Results:**

In total, 7.5%, 4.6% and 2.0% of the nurses included in the present study reported prescribed sleep medication, over‐the‐counter sleep medication or melatonin use in the last year, respectively. Short sleep duration, sleep problems and psychological conditions were strongly associated with both prescribed and over‐the‐counter sleep medication use. Nurses who worked more than 60 night shifts in the last year were at increased risk of sleep medication use.

## 1 INTRODUCTION

Many nurses are engaged in shift work which involves irregular or unusual working hours outside the normal daytime work schedule (Wang et al., 2011). Shift work, in particular night work, early morning shifts and less than 11 hr between shifts (quick returns), disrupts the normal sleep‐wake cycle and contributes to sleep disturbances and insufficient sleep (Kecklund & Axelsson, 2016). While many seem to tolerate shift work well, some do not. In this realm, shift work disorder (SWD) refers to symptoms of insomnia and/or sleepiness due a work schedule overlapping with the habitual sleep time and which is associated with a reduction of total sleep time (Waage et al., 2009). In a large epidemiological study among Norwegian nurses, the prevalence of SWD ranged between 6.2% and 40.7% depending on work time schedule (Flo et al., 2012). Shift workers are also at increased risk of developing insomnia (Kecklund & Axelsson, 2016). Both SWD and insomnia have detrimental effects on health. A recent meta‐analysis of longitudinal studies on shift work and mental health found an increased risk of depressive symptoms in shift workers, particularly in women (Torquati et al., 2019). Moreover, a recent Mendelian randomization study found evidence of a causal effect of insomnia on depression, diabetes and cardiovascular disease (Jansen et al., 2019).

A high prevalence of sleep problems in nurses is well documented (Bjorvatn et al., 2012; Eldevik et al., 2013; Flo et al., 2012), but less is known about how nurses cope with these problems. Treatments for sleep problems include sleep hygiene, cognitive behavioural therapy, and pharmacotherapy. In terms of the latter a range of pharmacological sleep aids are used including benzodiazepines, nonbenzodiazepines (z‐hypnotics), antihistamines, antidepressants, and neuroleptics with sedative effects. In Norway, the most commonly prescribed sleep medications are z‐hypnotics with zopiclone as the most frequently used type (Sakshaug et al., 2017). Beneficial effect of short‐term use of z‐hypnotics for insomnia has been reported (Huedo‐Medina et al., 2012), but in general, long‐term use is not recommended due to drug tolerance, risk of addiction and other adverse effects. In the guidelines for the diagnosis and treatment of insomnia, the European Sleep Research Society recommends cognitive behavioural therapy as the first‐line choice for treatment (Riemann et al., 2017). In persons aged 55 years or older, melatonin prolonged‐release tablets are indicated in treatment of insomnia. Melatonin is used as a sleep regulating medication which does not result in drug tolerance or addiction but is not recommended for treatment of insomnia in younger individuals (Riemann et al., 2017). Melatonin has until recently (1st of January 2020) only been available on prescription in Norway. Regarding sleep problems due to shift work, a recent Cochrane review found only low‐quality evidence for melatonin to improve sleep duration after shifts, while insufficient evidence was found for other sleep medications (Liira et al., 2014).

## 2 BACKGROUND

In the general population, prevalence rates for chronic and current use of sleep medication are commonly estimated to be between 4% and 10% (Ohayon & Lader, 2002; Omvik et al., 2010; Sakshaug et al., 2017). Sleep medication use has been found to be associated with older age, female sex, low socioeconomic status, and symptoms of sleep disorders, anxiety and depression (Ohayon & Lader, 2002; Omvik et al., 2010). Few studies have explored the prevalence of sleep medication use in a working population, and in nurses particularly, even though such use may have important implications on nurses’ health and well‐being. Since sleep medication use can result in drowsiness, it may also affect nurses’ performance and constitute a safety risk for themselves and their patients (Riemann et al., 2017). Among the studies that have been conducted during the last decade, the prevalence of sleep medication use in nurses has varied between 2% and 56% (Bjorvatn et al., 2012; Francis et al., 2019; Futenma et al., 2015; Shy et al., 2011). Studies that have explored the association between work time schedule and sleep medication use have shown conflicting results (Futenma et al., 2015; Gomez‐Garcia et al., 2016; Jensen et al., 2018; Sveinsdottir, 2006). In a recent Japanese cross‐sectional study (Futenma et al., 2015), the risk of sleep medication use was higher in nurses working day and night compared to nurses working day, night or evening, while a higher risk of sleep medication use in nurses working night shifts compared to nurses working day or rotating shifts was found in a Spanish cross‐sectional study (Gomez‐Garcia et al., 2016). A recent Danish and an Icelandic study, both cross‐sectional, found no difference in sleep medication use when comparing nurses with and without night work (Jensen et al., 2018; Sveinsdottir, 2006). Only one of the above‐mentioned studies have also investigated the association between use of hypnotics and demographic and health factors and found use to be increased in nurses aged ≥27 years old, and in nurses with symptoms of depression, SWD and insomnia (Futenma et al., 2015). Neither of these studies included information about over‐the‐counter sleep medication or melatonin use although these are commonly used sleep aids in the general population.

A better understanding of the prevalence and predictors of sleep medication use in nurses can identify risk factors, facilitate treatment of sleep problems and improve health. Against this backdrop, the aim of the present study was twofold; first to explore whether work‐related factors (shift work schedule, number of night shifts and quick returns the last year) were associated with prescribed sleep medication, over‐the‐counter sleep medication and melatonin use (separately) when controlling for age, sex, marital status, children living at home, work experience as a nurse, hours worked per week and type of workplace. And secondly, to explore whether indications of sleep problems (short sleep duration, sleepiness, shift work disorder and insomnia) or mental health problems (anxiety and depression) were associated with sleep medication and melatonin use independent of these work‐related factors. We hypothesized that nurses with a work schedule including night work, or with a higher number of night shifts or quick returns in the last year would have a higher risk of sleep medication and melatonin use than those without night work or a lower numbers of night shifts and quick returns. Further, we hypothesized that the presence of sleep problems and mental health problems would be associated with increased risk of sleep medication and melatonin use, independent of these work‐related factors.

## 3 METHOD

### 3.1 Study design

This was a cross‐sectional study.

### 3.2 Setting and participants

**FIGURE 1:**
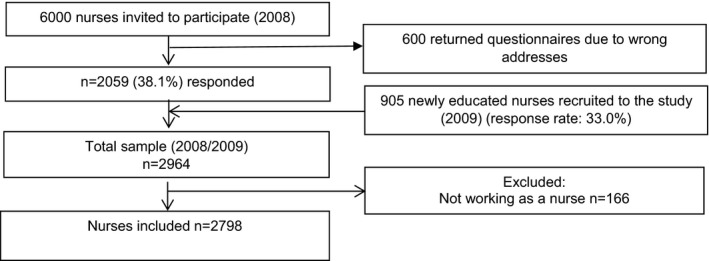
Selection of study sample

The present study was based on data from the SUrvey of Shift work, Sleep and Health (SUSSH) of Norwegian nurses. SUSSH was initiated in 2008 when a stratified sample of 6,000 nurses registered as members of the Norwegian Nurses Organization – a national labour union for nurses encompassing about 83% of all nurses in Norway (Søbstad et al., 2020) – was invited to participate in a survey. The sample was based on random sampling within stratas divided by time elapsed since graduation (less than 12 months, 1–3 years, >3–6 years, >6–9 years and >9–12 years). This sampling method was chosen to obtain sufficient variation in length of work experience among the nurses in the study sample. Of the 6,000 questionnaires that were sent out, 600 were returned due to wrong addresses. Of the 5,400 eligible nurses, 2059 consented to participate (response rate 38.1%). In 2009, an additional random sample of 905 newly graduated nurses (graduated June 2009) was recruited (response rate 33.0%) to increase the study population. The total sample therefore included 2,964 nurses. All included nurses responded to a postal questionnaire with questions related to demographic factors, work, health and lifestyle behaviour. We excluded 166 nurses who reported that they were not working at time of completing the questionnaire. This resulted in an analytic sample of 2,798 nurses (Figure 1).

### 3.3 Independent variables

To assess the association between work‐related factors and sleep medication and melatonin use, we included shift work schedule, number of night shifts and quick returns (“0,” “1–20,” “21–40,” “41–60” and “>60”) as work‐related predictors, as these variables are particularly relevant for the working conditions we wanted to study. The categories for night shifts and quick returns were chosen to obtain approximately equally sized groups. We collected detailed information about shift work schedule, but due to small groups, “permanent day work,” “only evening work” and “2‐shift (day and evening)” were categorized as “schedule without night work,” and “only night work,” “3‐shift (day, evening and night)” and “other schedule including night work” were categorized as “schedule with night work.” Based on experience from clinical practice and previous research, we included measures related to sleep and mental health as well as background variables that we a priori expected to be associated with sleep medication and melatonin use (Futenma et al., 2015; Omvik et al., 2010; S. Waage et al., 2014). This included sleep duration (“<6 hr,” “6‐<7 hr,” “7‐<8 hr,” “8–9 hr,” “>9 hr”), excessive daytime sleepiness, shift work disorder (SWD), and insomnia as measures of sleep, and anxiety and depression as measures of mental health. Registered background variables included sex, age (included as a continuous variable), marital status (“partner,” “single”), children living at home (“yes,” “no”), number of years of experience working as a nurse (included as a continuous variable, less than 6 months were reported as 0 years of experience), average hours worked per week (continuous variable) and type of workplace (“acute care” (somatic and psychiatric hospital), “community‐based care” (nursing home, home‐based care or healthcare centre)). A total of 108 nurses had reported “other” on type of workplace – this category was coded missing.

#### 3.3.1 Instruments

The questionnaire included several validated assessment tools. Insomnia (yes, no) was measured by the Bergen Insomnia Scale (BIS) (Pallesen et al., 2008). The BIS asks how many days per week during the last month one has experienced problems related to sleep, and daytime consequences. If the nurse reported three or more days on at least one of the first four items (nocturnal problems), and three or more days on at least one of the last two items (daytime consequences), insomnia was coded 1 (“yes”) according to the inclusion criteria for insomnia found in the fourth edition of the Diagnostic and Statistical Manual of Mental Disorders (American Psychiatric Association, 1994). Cronbach's alpha for the BIS was 0.83 in the present study.

SWD was assessed using three questions previously developed for use in epidemiological studies: 1) Do you experience difficulties with sleeping or excessive sleepiness? 2) Is the sleep or sleepiness problem related to a work schedule where you have to work when you would normally sleep? 3) Has this sleep or sleepiness problem related to your work schedule persisted for at least one month? (Waage et al., 2009) These items adhered to the diagnostic criteria for SWD found in the second edition of the International Classification of Sleep Disorders (ICSD‐2) (American Academy of Sleep Medicine, 2005). SWD was coded 1 (“yes”) if the responded answered “yes” to all three questions.

Epworth Sleepiness Scale (ESS) (Johns, 1991) is a standardized subjective measure of chronic sleepiness that has been validated for use in Norwegian (Pallesen et al., 2007). The respondents are presented with eight different situations and asked to estimate the likelihood of dozing off in each situation using a 4‐point scale (0 = no risk of dozing off, 3 = high risk of dozing off). A compositive score is calculating by adding the score of each item. A composite score of 11 or higher is suggestive of excessive sleepiness. Cronbach's alpha for the ESS in the present study was 0.74.

The Hospital Anxiety and Depression Scale (HADS) was used to measure symptoms of anxiety and depression (Zigmond & Snaith, 1983). A validated Norwegian version of HADS was used (Bjelland et al., 2002). HADS consists of 14 items, of which 7 measures non‐vegetative symptoms of anxiety and 7 measures non‐vegetative symptoms of depression. The respondents score each item from 0 to 3, and a sum score for each subscale is created by adding the score of each item. In accordance with previous studies, a cut‐off of 8 or above was used for categorizing anxiety and depression, respectively (Bjelland et al., 2002). In the present study, Cronbach's alpha for the anxiety and depression subscale was 0.81 and 0.82, respectively.

### 3.4 Outcome variables

The nurses were asked whether they during the last year had used any of the following: 1) prescribed, or 2) over‐the‐counter sleep medication, or 3) melatonin (“yes,” “no”).

### 3.5 Analysis

We compared the prevalence of prescribed and over‐the‐counter sleep medication and melatonin use by categories of the predictors using Pearson's chi‐square test. In this analysis, continuous variables were categorized, to make it possible to compare the prevalence of the outcomes in different groups. Age was categorized as “21–25 years old,” “26–30,” “31–35”, “36–40” and “>40,” work experience as “<1 year,” “1–2 years,” “3–5,” “6–10” and “>10,” and average hours worked per week as “≤20 hr,” “20.1–30,” “30.1–37.5,” “37.6–40” and “>40.” We used a modified Poisson model to estimate crude and adjusted relative risks (RR) with 95% confidence intervals (CI) for the association between each predictor and each of the three outcome variables (Zou, 2004). We first ran separate models to estimate the association between each of the three work‐related factors (work time schedule, number of night shifts and quick returns) and sleep medication and melatonin use, adjusting for sex, age, marital status, children living at home, years of experience, hours worked per week and type of workplace. Then, we estimated one model for each of the factors related to sleep or mental health (including sleep duration, ESS, SWD, insomnia, symptoms of anxiety and depression) adjusting for the same background variables, in addition to the four work‐related factors. Since we were interested in the independent association between each of the sleep and mental health factors and sleep medication and melatonin use, work‐related factors were adjusted for as these could affect both the degree of these problems and sleep medication and melatonin use. For models that included a continuous independent variable, we tested for departure from linearity in associations between the independent variable and each of the three outcomes by comparing a model with and without a quadratic term using a likelihood‐ratio test. We also explored whether the association between SWD, insomnia, excessive sleepiness, anxiety and depression and each of the three outcome variables varied according to work time schedule (with or without night work), number of night shifts or quick returns, by including an interaction term in the model, also investigated by a likelihood‐ratio test. All statistical analyses were conducted in Stata/SE version 16.0 (StataCorp, College Station, TX, USA).

### 3.6 Ethics

This study was approved by the Regional Committee for Medical Research Ethics in Western Norway (No. 088.88). Informed consent was obtained from all participants.

## 4 RESULTS

The study sample included 2,522 women (90.2%) and 274 men (9.8%) (2 nurses had missing data on sex). The mean age was 31.9 years and 28.0% had less than one year of work experience (Table 1). A total of 68.7% of the nurses had a partner, and 41.1% reported to have children living at home. The majority worked a schedule that included night work (64.2%), and 9.2% and 12.9% of the nurses reported to have worked more than 60 night shifts or quick returns, respectively, during the last year. A large majority of the nurses (83.8%) worked within acute care. In total, 34.7% of the nurses reported SWD and 52.0% insomnia, and 27.3% reported symptoms of excessive daytime sleepiness.

**TABLE 1:** Distribution of predictors among the nurses (*N* = 2,798) included in the study sample

Variable	*N* (%) or mean (*SD*)
Sex, *N* = 2,796 (%)	
Female	2,522 (90.2)
Male	274 (9.8)
Age (years), *N* = 2,790, mean (*SD*)	31.9 (8.4)
Marital status, *N* = 2,771 (%)	
Partner	1904 (68.7)
Single	867 (31.3)
Children living at home, *N* = 2,669 (%)	
Yes	1,096 (41.1)
No	1573 (58.9)
Work experience (years), *N* = 2,764, mean (*SD*)	3.7 (4.3)
Hours worked per week, *N* = 2,745, mean (*SD*)	33.7 (7.1)
Type of workplace, *N* = 2,631 (%)	
Acute care	2,204 (83.8)
Community‐based care	427 (16.3)
Shift work schedule, *N* = 2,759 (%)	
Schedule without night work	988 (35.8)
Schedule with night work	1771 (64.2)
Number of night shifts, *N* = 2,755 (%)	
0	693 (25.2)
1–20	936 (34.0)
21–40	617 (22.4)
41–60	255 (9.3)
>60	254 (9.2)
Number of quick returns, *N* = 2,660 (%)	
0	311 (11.7)
1–20	890 (33.5)
21–40	572 (21.5)
41–60	545 (20.5)
>60	342 (12.9)
Sleep duration, *N* = 2,780, mean (*SD*)	
<6 hr	167 (6.0)
6‐<7 hr	797 (28.7)
7‐<8 hr	1,185 (42.6)
8–9 hr	606 (21.8)
>9 hr	25 (0.9)
Excessive daytime sleepiness, *N* = 2,789 (%)	
No (ESS score <11)	2028 (72.7)
Yes (ESS score ≥11)	761 (27.3)
Shift work disorder (SWD), *N* = 2,782 (%)	
No	1817 (65.3)
Yes	965 (34.7)
Insomnia, *N* = 2,794 (%)	
No	1,342 (48.0)
Yes	1,452 (52.0)
Anxiety, *N* = 2,785 (%)	
No (HADS‐A score <8)	2,223 (79.8)
Yes (HADS‐A score ≥8)	562 (20.2)
Depression, *N* = 2,785 (%)	
No (HADS‐D score <8)	2,541 (91.2)
Yes (HADS‐D score ≥8)	244 (8.8)

In all, 210 (7.5%, 95% confidence interval (CI) 6.6%–8.5%) reported prescribed sleep medication use, while 130 (4.6%, 95% CI 3.9%–5.5%) and 56 (2.0%, 95% CI 1.5%–2.6%) reported over‐the‐counter sleep medication or melatonin use during the last year, respectively (Table 2).

**TABLE 2:** Prevalence of sleep medication (prescribed and over the counter) and melatonin use by independent variables among Norwegian nurses (*N* = 2,798)

Independent variables	Prescribed sleep medication (*N* = 210, 7.5%)		Sleep medication over the counter (*N* = 130, 4.6%)		Melatonin (*N* = 56, 2.0%)	
	% (*N*)	*p*‐value^a^	% (*N*)	*p*‐value^a^	% (*N*)	*p*‐value^a^
Sex						
Female	7.6 (191)	.703	4.7 (118)	.619	1.9 (47)	.111
Male	6.9 (19)		4.0 (11)		3.3 (9)	
Age (years)						
21–25	3.9 (30)	<.001	3.2 (25)	.081	1.2 (9)	.001
26–30	6.0 (42)		5.0 (35)		2.0 (14)	
31–35	7.5 (41)		4.4 (24)		1.3 (7)	
36–40	8.7 (28)		5.0 (16)		1.9 (6)	
>40	15.5 (69)		6.7 (30)		4.5 (20)	
Marital status						
Partner	6.7 (128)	.015	4.6 (87)	.652	1.6 (31)	.029
Single	9.3 (81)		5.0 (43)		2.9 (25)	
Children living at home						
Yes	6.6 (72)	.222	3.4 (37)	.011	2.5 (27)	.177
No	7.8 (123)		5.5 (86)		1.7 (27)	
Work experience (years)						
<1	5.1 (39)	.003	3.2 (25)	.012	1.7 (13)	.914
1–2	7.2 (53)		5.0 (37)		2.2 (16)	
3–5	7.7 (37)		6.2 (30)		2.1 (10)	
6–10	9.1 (54)		3.9 (23)		2.4 (14)	
>10	12.9 (23)		8.4 (15)		1.7 (3)	
Average hours worked per week						
≤20	9.4 (15)	.119	5.0 (8)	.736	1.3 (2)	.158
20.1–30	8.8 (56)		4.9 (31)		2.7 (17)	
31.1–37.5	6.2 (85)		4.2 (57)		1.5 (21)	
37.6–40	8.4 (37)		5.7 (25)		3.2 (14)	
>40	5.2 (7)		5.2 (7)		1.5 (2)	
Type of workplace						
Acute care	171 (7.8)	.303	115 (5.2)	.019	42 (1.9)	.965
Community‐based care	27 (6.3)		11 (2.6)		8 (1.9)	
Shift work schedule						
Schedule without night work	7.9 (78)	.487	4.5 (44)	.680	2.5 (25)	.132
Schedule with night work	7.2 (127)		4.8 (85)		1.7 (30)	
Number of night shifts						
0	8.7 (60)	.001	3.8 (26)	.281	2.5 (17)	.180
1–20	6.8 (64)		4.6 (43)		2.0 (19)	
21–40	5.7 (35)		5.4 (33)		1.6 (10)	
41–60	5.9 (15)		3.5 (9)		0.4 (1)	
>60	13.4 (34)		6.7 (17)		3.2 (8)	
Number of quick returns						
0	10.0 (31)	.083	3.5 (11)	.631	2.3 (7)	.855
1–20	5.8 (52)		4.4 (39)		1.8 (16)	
21–40	6.5 (37)		5.1 (29)		1.9 (11)	
41–60	8.1 (44)		5.7 (31)		1.7 (9)	
>60	8.8 (30)		4.4 (15)		2.6 (9)	
Sleep duration						
<6 hr	21.6 (36)	<.001	11.4 (19)	<.001	7.8 (13)	<.001
6‐<7 hr	9.2 (73)		6.3 (50)		2.5 (20)	
7‐<8 hr	5.7 (67)		3.5 (42)		1.3 (15)	
8–9 hr	5.1 (31)		2.8 (17)		1.3 (8)	
>9 hr	8.0 (2)		4.0 (1)		0.0 (0)	
Excessive daytime sleepiness						
No	7.2 (146)	.280	4.2 (85)	.055	1.9 (38)	.410
Yes	8.4 (64)		5.9 (45)		2.4 (18)	
Shift work disorder (SWD)						
No	4.9 (85)	<.001	2.4 (44)	<.001	1.2 (22)	<.001
Yes	13.0 (125)		8.9 (86)		3.5 (34)	
Insomnia						
No	2.5 (34)	<.001	1.7 (23)	<.001	0.5 (7)	<.001
Yes	12.1 (176)		7.4 (107)		3.4 (49)	
Anxiety						
No	5.4 (119)	<.001	3.6 (80)	<.001	1.5 (33)	<.001
Yes	16.2 (91)		8.7 (49)		4.1 (23)	
Depression						
No	6.4 (162)	<.001	4.3 (109)	.006	1.9 (47)	.051
Yes	19.7 (48)		8.2 (20)		3.7 (9)	

^a^ *p*‐value from a Pearson's chi‐squared test.

In the adjusted regression models for the work‐related predictors, we only found an association between number of night shifts last year and prescribed or over‐the‐counter sleep medication use (Table 3). Nurses who had worked more than 60 night shifts last year, had approximately a 2‐fold increased risk of prescribed and over‐the‐counter sleep medication use compared to nurses who worked no night shifts last year (adjusted RR (aRR) 1.75, 95% CI 1.12–2.73 for prescribed sleep medication and aRR 1.99, 95% CI 1.05–3.75 for over‐the‐counter sleep medication) (Table 3). Risk of prescribed and over‐the‐counter sleep medication and melatonin use increased with increasing age (Table 3). Further, single nurses had an increased risk of prescribed sleep medication and melatonin use compared to nurses with partners (aRR 1.59, 95% CI 1.16–2.18 for prescribed sleep medication and aRR 2.25, 95% CI 1.23–4.14 for melatonin). Nurses without children living at home had an increased risk of prescribed sleep medication and over‐the‐counter sleep medication use compared to nurses with children at home (aRR 1.53, 95% CI 1.10–2.14 for prescribed and aRR 2.29, 95% CI 1.52–3.44 for over‐the‐counter sleep medication). A lower risk of over‐the‐counter sleep medication use was found for nurses working within community‐based care compared to acute care, but this association was no longer statistically significant in the adjusted analysis (aRR 0.59, 95% CI 0.31–1.10).

**TABLE 3:** Crude and adjusted relative risk of sleep medication (prescribed or over the counter) and melatonin use by demographic and work‐related factors among Norwegian nurses (*N* = 2,798)

Independent variable	Prescribed sleep medication		Over‐the‐counter sleep medication		Melatonin	
	Crude RR (95% CI)	Adjusted RR^a^ (95% CI)	Crude RR (95% CI)	Adjusted RR^a^ (95% CI)	Crude RR (95% CI)	Adjusted RR^a^ (95% CI)
Sex						
Female	1.00 (ref.)	1.00 (ref.)	1.00 (ref.)	1.00 (ref.)	1.00 (ref.)	1.00 (ref.)
Male	0.92 (0.58–1.44)	1.06 (0.66–1.70)	0.86 (0.47–1.57)	0.85 (0.45–1.62)	1.76 (0.87–3.56)	2.09 (1.01–4.34)
Age (years)	1.06 (1.04–1.07)	1.06 (1.05–1.08)	1.02 (1.01–1.04)	1.03 (1.01–1.05)	1.05 (1.02–1.08)	1.06 (1.03–1.10)
Marital status						
Partner	1.00 (ref.)	1.00 (ref.)	1.00 (ref.)	1.00 (ref.)	1.00 (ref.)	1.00 (ref.)
Single	1.39 (1.06–1.81)	1.59 (1.16–2.18)	1.09 (0.76–1.55)	0.94 (0.63–1.40)	1.77 (1.05–2.98)	2.25 (1.23–4.14)
Children living at home						
Yes	1.00 (ref.)	1.00 (ref.)	1.00 (ref.)	1.00 (ref.)	1.00 (ref.)	1.00 (ref.)
No	1.19 (0.90–1.58)	1.53 (1.10–2.14)	1.62 (1.11–2.36)	2.29 (1.52–3.44)	0.70 (0.41–1.18)	0.80 (0.40–1.58)
Work experience (years)	1.05 (1.03–1.08)	1.01 (0.98–1.03)	1.04 (1.01–1.07)	1.01 (0.98–1.06)	1.01 (0.96–1.06)	0.98 (0.92–1.03)
Average hours worked per week	0.99 (0.97–1.01)	0.99 (0.97–1.01)	0.99 (0.97–1.02)	0.98 (0.96–1.01)	1.01 (0.97–1.06)	1.01 (0.96–1.06)
Type of workplace						
Acute care	1.00 (ref.)	1.00 (ref.)	1.00 (ref.)	1.00 (ref.)	1.00 (ref.)	1.00 (ref.)
Community‐based care	0.81 (0.55–1.21)	1.00 (0.66–1.52)	0.49 (0.27–0.91)	0.59 (0.31–1.10)	0.98 (0.46–2.08	1.11 (0.51–2.42)
Shift work schedule						
Without night work	1.00 (ref.)	1.00 (ref.)	1.00 (ref.)	1.00 (ref.)	1.00 (ref.)	1.00 (ref.)
With night work	0.90 (0.68–1.97)	1.11 (0.81–1.52)	1.10 (0.76–1.58)	1.07 (0.72–1.59)	0.67 (0.39–1.13)	0.72 (0.38–1.36)
Number of night shifts						
0	1.00 (ref.)	1.00 (ref.)	1.00 (ref.)	1.00 (ref.)	1.00 (ref.)	1.00 (ref.)
1–20	0.79 (0.56–1.11)	1.01 (0.70–1.47)	1.22 (0.76–1.97)	1.34 (0.80–2.24)	0.83 (0.43–1.58)	0.81 (0.41–1.60)
21–40	0.66 (0.44–0.98)	0.85 (0.55–1.31)	1.43 (0.86–2.36)	1.31 (0.75–2.29)	0.66 (0.34–1.43)	0.68 (0.31–1.51)
41–60	0.68 (0.39–1.17)	0.96 (0.54–1.71)	0.94 (0.45–1.98)	1.07 (0.50–2.31)	0.16 (0.02–1.20)	0.19 (0.03–1.34)
>60	1.55 (1.04–2.30)	1.75 (1.12–2.73)	1.78 (0.98–3.23)	1.99 (1.05–3.75)	1.28 (0.56–2.94)	0.93 (0.34–2.55)
Number of quick returns						
0	1.00 (ref.)	1.00 (ref.)	1.00 (ref.)	1.00 (ref.)	1.00 (ref.)	1.00 (ref.)
1–20	0.59 (0.38–0.90)	0.84 (0.52–1.36)	1.24 (0.64–2.39)	1.57 (0.76–3.25)	0.80 (0.33–1.92)	1.09 (0.41–2.94)
21–40	0.65 (0.41–1.02)	1.03 (0.62–1.71)	1.43 (0.73–2.83)	1.61 (0.76–3.39)	0.85 (0.33–2.18)	0.93 (0.31–2.77)
41–60	0.81 (0.52–1.26)	1.18 (0.72–1.91)	1.61 (0.82–3.15)	1.64 (0.77–3.49)	0.73 (0.28–1.95)	0.93 (0.30–2.86)
>60	0.88 (0.54–1.42)	1.06 (0.62–1.83)	1.24 (0.58–2.66)	1.15 (0.49–2.69)	1.17 (0.44–3.10)	1.36 (0.44–4.27)

^a^ Adjusted for sex, age, marital status, children living at home, work experience, hours worked per week and type of workplace.

Prescribed and over‐the‐counter sleep medication use were strongly associated with short sleep duration (less than 7 hr), SWD, insomnia, and symptoms of depression and anxiety when adjusting for all background variables as well as the work‐related factors (Table 4). Short sleep duration of less than 6 hr, SWD, insomnia and anxiety were also strongly associated with melatonin use. We found a statistically significant interaction effect (*p* = .05) between SWD and work schedule for the association with prescribed sleep medication use; the association between prescribed sleep medication use and SWD was stronger for nurses with a work schedule including night work than for nurses with a schedule not including night work.

**TABLE 4:** Crude and adjusted relative risk of sleep medication (prescribed or over the counter) and melatonin use by indication of sleep and mental health factors among Norwegian nurses (*N* = 2,798)

Independent variables	Prescribed sleep medication		Over‐the‐counter sleep medication		Melatonin	
	Crude RR (95% CI)	Adjusted RR^a^ (95% CI)	Crude RR (95% CI)	Adjusted RR^a^ (95% CI)	Crude RR (95% CI)	Adjusted RR^a^ (95% CI)
Sleep duration						
<6 hr	3.81 (2.63–5.53)	3.66 (2.41–5.56)	3.21 (1.91–5.38)	3.62 (2.08–6.29)	6.15 (2.98–12.70)	4.45 (1.91–10.39)
6‐<7 hr	1.62 (1.18–2.23)	1.44 (1.01–2.04)	1.77 (1.19–2.64)	1.71 (1.10–2.65)	1.98 (1.02–3.85)	2.02 (0.96–4.28)
7‐<8 hr	1.00 (ref.)	1.00 (ref.)	1.00 (ref.)	1.00 (ref.)	1.00 (ref.)	1.00 (ref.)
8–9 hr	0.90 (0.60–1.37)	0.81 (0.51–1.29)	0.79 (0.45–1.38)	0.73 (0.40–1.33)	1.04 (0.44–2.45)	1.31 (0.52–3.31)
>9 hr	1.41 (0.37–5.46)	1.65 (0.44–6.14)	1.13 (0.16–7.88)	1.13 (0.15–8.29)	N/A	N/A
Excessive sleepiness						
No	1.00 (ref.)	1.00 (ref.)	1.00 (ref.)	1.00 (ref.)	1.00 (ref.)	1.00 (ref.)
Yes	1.17 (0.88–1.55)	1.20 (0.88–1.64)	1.41 (0.99–2.01)	1.36 (0.93–1.99)	1.26 (0.72–2.20)	0.96 (0.49–1.87)
Shift work disorder (SWD)						
No	1.00 (ref.)	1.00 (ref.)	1.00 (ref.)	1.00 (ref.)	1.00 (ref.)	1.00 (ref.)
Yes	2.77 (2.13–3.61)	2.91 (2.14–3.95)	3.68 (2.58–5.25)	3.70 (2.49–5.51)	2.91 (1.71–4.95)	3.20 (1.78–5.75)
Insomnia						
No	1.00 (ref.)	1.00 (ref.)	1.00 (ref.)	1.00 (ref.)	1.00 (ref.)	1.00 (ref.)
Yes	4.78 (3.34–6.86)	5.44 (3.55–8.32)	4.30 (2.76–6.71)	4.09 (2.55–6.54)	6.47 (2.94–14.24)	6.98 (2.75–17.71)
Anxiety						
No	1.00 (ref.)	1.00 (ref.)	1.00 (ref.)	1.00 (ref.)	1.00 (ref.)	1.00 (ref.)
Yes	3.02 (2.34–3.91)	2.93 (2.19–3.91)	2.42 (1.72–3.42)	2.32 (1.59–3.37)	2.76 (1.63–4.66)	2.38 (1.29–4.40)
Depression						
No	1.00 (ref.)	1.00 (ref.)	1.00 (ref.)	1.00 (ref.)	1.00 (ref.)	1.00 (ref.)
Yes	3.09 (2.30–4.14)	2.74 (1.94–3.85)	1.91 (1.21–3.02)	1.95 (1.17–3.23)	1.99 (0.99–4.02)	1.45 (0.63–3.36)

Note

N/A: No cases in exposure group.

^a^ Adjusted for sex, age, marital status, children living at home, work experience, hours worked per week, type of workplace, shift work schedule, number of night shifts and quick returns.

## 5 DISCUSSION

The aim of this cross‐sectional study comprising 2,798 Norwegian nurses was to estimate the prevalence of sleep medication and melatonin use and to identify factors associated with such use. The association between shift work and sleep problems in nurses is well documented, but few studies have explored the use of different pharmacological sleep aids in nurses. By including data on both prescribed and over‐the‐counter sleep medication as well as melatonin use, we provide a more complete overview of use than in previous studies. In total, 7.5% of the nurses reported prescribed sleep medication use the last year, while 4.7% and 2.0% reported over‐the‐counter sleep medication and melatonin use, respectively. Prevalence of sleep medication use in nurses in previous studies has varied greatly, ranging from 2%–56% (Bjorvatn et al., 2012; Francis et al., 2019; Futenma et al., 2015; Jensen et al., 2018; Shy et al., 2011). Comparison with previous studies is however problematic due to differences in working time arrangements, operationalization of sleep medication use, sample characteristics and time frame across studies. Very few of the previous studies have included information on over‐the‐counter sleep medication and melatonin use. The proportion of nurses reporting prescribed sleep medication use the last year in the present study was similar to what has been reported in recent studies based on representative samples of the Norwegian population (Omvik et al., 2010; Sakshaug et al., 2017). Hence, the prevalence of prescribed sleep medication use found among nurses in SUSSH seems not to be higher than what has been reported in the general population.

Contrary to what we hypothesized, we did not find any association between work schedule (schedule with or without night work) and number of quick returns and sleep medication or melatonin use. However, we found a higher risk of prescribed and over‐the‐counter sleep medication use in nurses with more than 60 night shifts last year. Shift work, especially night work, and quick returns, have been found to be associated with increased risk of SWD and insomnia (Eldevik et al., 2013; Øyane et al., 2013). The lack of an observed increased risk in sleep medication use in nurses with a work schedule with night work or a high number of quick returns in the present study could partly be explained by the “healthy worker effect” (Knutsson, 2004). This effect implies that nurses who have struggled the most with sleep problems due to night work and quick returns have left such work and switched to permanent day work before the study was initiated. Although sleep medication use could be related to other factors, it is likely that sleep medication use would be higher if there was no possibility to select out of night work or quick returns as such work schedules are strongly associated with sleep disturbances and insufficient sleep. The crude operationalization of shift work could explain the lack of an independent association between shift work schedule and sleep medication and melatonin use. Previous studies on work schedule and sleep medication use in nurses have reported conflicting results. Across studies, work schedule and sleep medication use have been defined and categorized in different ways. In a recent cross‐sectional study including 114 intensive care nurses at two hospitals in Denmark, no difference in sleep medication use was found between nurses with and without night work (Jensen et al., 2018). In addition to a small sample size, exposure to night work was poorly classified as all nurses in that study reported some night work. A recent cross‐sectional study from Japan including 997 female hospital nurses found the risk of sleep medication use to be higher in 2‐shift (working day and night) than in 3‐shift workers (working day, evening and night) (Futenma et al., 2015). However, as in the Danish study, both groups had a work schedule that included night work. In a cross‐sectional study including 394 Icelandic nurses who worked either permanent day, 2‐shift (day and evening), or 3‐shift (day, evening and night), there was no statistically significant difference in sleep medication use across groups (Sveinsdottir, 2006). In that study, the nurses were asked how often they had used sleep medication during the last 6 months. Another cross‐sectional study including 635 nurses working in seven hospitals in Spain measured sleep medication use based on an item in the Pittsburgh Sleep Quality Index (Gomez‐Garcia et al., 2016). That study reported a higher sleep medication use in nurses working only nights compared to nurses working day or rotating shifts. Based on these results and the results in the present study, the association between work‐related factors and sleep medication use remain unclear and should be further investigated, preferably by longitudinal designs initiated before the respondents start working as nurses.

In accordance with what we hypothesized, we found short sleep duration, and symptoms of SWD, insomnia, anxiety and depression to be strongly associated with sleep medication and melatonin use. This is also in accordance with the above‐mentioned Japanese study (Futenma et al., 2015). Adjustment for demographic and work‐related factors hardly affected the results. The observed associations between the sleep and mental health factors and the three outcome variables did not vary by work time schedule, number of night shifts or quick returns, except for SWD, in which the association with prescribed sleep medication use was stronger in nurses with a work schedule including night work compared to nurses without night work.

In the present sample, we found a high prevalence of SWD and insomnia (34.7% and 52.0%, respectively). A recent study with a representative sample of the Norwegian population, reported that 20.0% had symptoms of insomnia (Bjorvatn et al., 2018). In that study insomnia was defined with a time frame including the last 3 months based on the third edition of the International Classification of Sleep Disorders (ICSD‐3) diagnostic criteria (Sateia, 2014), while in the present study, we used the definition based on the previous edition, the ICSD‐2 (American Academy of Sleep Medicine, 2005). Still, the present study suggests that insomnia is very common among nurses.

Contrary to what we hypothesized, the difference in prevalence and risk of sleep medication and melatonin use for nurses with and without symptoms of excessive sleepiness was small and not statistically significant. Excessive sleepiness may be due to sleep problems (Boyes et al., 2017), and thereby expected to be associated with increased sleep medication use. On the other hand, prescribed sleep medication use may as well induce excessive daytime sleepiness, since drowsiness after the sleep period is a potential side effect (Liira et al., 2014). Due to the cross‐sectional design of the present study, we cannot assess the direction of this association. The prevalence of excessive daytime sleepiness was high in the present sample compared to what has been reported in the general Norwegian population; 27.3% compared to 17.7% in a sample of 2,301 Norwegian adults (Pallesen et al., 2007). None of the previous studies that investigated predictors of sleep medication use in nurses did however include a direct measure of excessive sleepiness.

Furthermore, in line with previous studies (Futenma et al., 2015), we found age to be positively associated with sleep medication and melatonin use. The role of other demographic factors, including marital status and children living at home, has been less explored. The lower risk found in nurses with partners or with children living at home could point towards better health in these nurses (Cañadas‐De la Fuente et al., 2018). Family obligations may make them more reluctant to use sleep medication due to possible adverse side effects, or that a busy family life contributes to more regular sleep habits. Factors related to type of workplace could be relevant predictors of sleep medication and melatonin use. We did find a lower risk of over‐the‐counter sleep medication use in nurses in community‐based care compared to nurses within acute care, but this association was partly explained by other variables in the model and did not remain statistically significant after adjustment. Future studies should consider including more detailed data on workplace characteristics such as workload, strain and social support.

The strengths of the present study include its large sample size and the fact that we separated between prescribed and over‐the‐counter sleep medication and melatonin use. In addition, we included different classifications of work‐related factors (work experience, work schedule, number of night shifts and quick returns), a broad set of demographic variables, and employed standardized and validated instruments for SWD, insomnia, sleepiness, anxiety and depression.

An important limitation of the present study entails the small number of nurses in the groups of some of the predictors leading to low statistical power in parts of the analyses. Another important limitation was the low response rate (mid‐thirties). This may have affected the internal validity and generalizability of the study. We do not know whether non‐responders were more or less likely to use sleep medication. Hence, it is difficult to assess how the low response rate might have affected the results. In general, non‐responders are often found to be of poorer health, but whether or not non‐response leads to biased results varies by type of health outcome studied (Knudsen et al., 2010; Langhammer et al., 2012; Vercambre & Gilbert, 2012). As a result of the low response rate, the reported overall prevalence of sleep medication and melatonin use should be interpreted with caution. In addition, the cohort is younger than the Norwegian nurse work force overall. This could lead to an underestimation of the true prevalence of sleep medication and melatonin use given that such use was found to be less prevalent among younger than older nurses in the present study. More than 90% of the nurses included in the cohort were female, this is, however, representative of the sex distribution of Norwegian nurses (Statistics Norway, 2019).

Other important limitations include the lack of information on type and amount of medication, frequency of use, and the fact that we only had self‐reported data on sleep medication use. Also, as sleep medication use by many is regarded as undesirable, the results may have been influenced by social desirability bias (Krumpal, 2013). Assessments of presence of sleep problems, mental health problems and hypnotic use were also exclusively based on self‐reported information; thus, we did not have any information from clinical interviews, clinical records or health registry data. We also do not know how the respondents interpreted the term “sleep medication” (e.g. in the case of antidepressants with sedative effects), and what type of sleep medication they used. The prevalence of sleep medication and melatonin use reported in the present study is not necessarily representative of the prevalence among nurses today given that our data were surveyed more than a decade ago. In the general Norwegian population, total sale of prescribed sleep medication, measured as dispensed defined daily doses, has decreased during the last decade, while the total sale of melatonin has increased (Sakshaug et al., 2019). Further, the association between sleep medication and melatonin use and the included predictors may have changed in the last decade, but in what direction is harder to predict. Hence, in summary, future studies should include newer and more detailed information of the specific medications used, preferably based on prescription registries. We do not know if these data are generalizable to other countries as work hours, workload and other relevant factors differ between countries. Similar studies in other countries are therefore warranted.

## 6 CONCLUSION

In total, 7.5%, 4.6% and 2.0% of the nurses included in the present study reported prescribed sleep medication, over‐the‐counter sleep medication and melatonin use during the last year, respectively. Short sleep duration, symptoms of SWD, insomnia, anxiety and depression were all strongly associated with both sleep medication (all) and melatonin (all except depression) use. These findings are overall in line with previous studies. No associations were found between work schedule and sleep medication or melatonin use, while prescribed and over‐the‐counter sleep medication use were more prevalent in nurses working more than 60 night shifts the last year. Sleep medication use may have important implications on nurses’ health, well‐being and performance and deserve further exploration. Future studies should explore the long‐term effects of cumulative exposure to shift work on sleep medication use with longitudinal designs, and should also include data on shifts, mental disorders and medication use from sources such as health registries.

## 7 RELEVANCE TO CLINICAL PRACTICE

Special attention should be paid to nurses working a high number of night shifts, and to nurses with indications of sleep problems or anxiety/depression, independent of work time schedule. Longitudinal studies are needed to assess the long‐term effect of night work on sleep medication and melatonin use.

## CONFLICT OF INTEREST

The authors declare that they have no conflict of interest.

## AUTHOR CONTRIBUTIONS

All authors contributed to the conception and design of the study. SW, SP, BEM and BB contributed to the acquisition of data. IF conducted the analyses and drafted the first draft of the manuscript. All authors contributed to the interpretation of the data, and reviewed, critically revised, and approved the final version of the manuscript. All authors agree to be accountable for all aspects of the work.

## Data Availability

The data that support the findings of this study are available from the corresponding author upon reasonable request.
